# Phagocytosis and neutrophil extracellular traps

**DOI:** 10.12703/r/9-25

**Published:** 2020-12-21

**Authors:** Frank R DeLeo, Lee-Ann H Allen

**Affiliations:** 1 Laboratory of Bacteriology, Rocky Mountain Laboratories, National Institute of Allergy and Infectious Diseases, National Institutes of Health, Hamilton, MT, USA; 2 Inflammation Program, Department of Internal Medicine and Department of Microbiology and Immunology, University of Iowa, Iowa City, IA, USA; 3 VA Healthcare System, Iowa City, IA, USA; 4 Department of Molecular Microbiology and Immunology, University of Missouri School of Medicine, Columbia, MO, USA

**Keywords:** Neutrophil, neutrophil extracellular trap, NETs, phagocytosis

## Abstract

Neutrophils are recruited rapidly to sites of infection in response to host- and/or microbe-derived proinflammatory molecules. At such sites, neutrophils phagocytose microbes and are activated to produce superoxide and other reactive oxygen species (ROS). In addition, neutrophils contain stores of antimicrobial peptides and enzymes that work in concert with ROS to kill ingested microbes. Neutrophils can also release chromosomal DNA bound with antimicrobial peptides and enzymes to form web-like structures known as extracellular traps. Neutrophil extracellular traps (NETs) have been reported to ensnare and kill microbes and are commonly considered to be an important component of innate host defense. Notably, the formation of NETs is most often reported as a cytolytic process. Whereas intraphagosomal killing of microbes sequesters cytotoxic antimicrobial molecules that would otherwise damage host tissues, the formation of NETs and associated extracellular release of these molecules can contribute to host tissue destruction and disease. Here we compare and contrast phagocytosis and NETs in host defense, with emphasis on recent studies of NETs that ultimately underscore the importance of phagocytosis as the primary means by which neutrophils eliminate microbes.

## Introduction

Neutrophils are the most abundant leukocytes in human blood, and their central role in innate defense against infection is unequivocal. The processes that recruit neutrophils to sites of microbe invasion, phagocytosis, and coupling of the engulfment process to rapid deployment of oxygen-dependent and oxygen-independent mechanisms for efficient microbe killing and digestion have been studied extensively. Many fundamental insights into these neutrophil processes have arisen from studies of human patients and bacterial and fungal pathogens to inhibit or coopt these defense processes to evade elimination while also causing tissue destruction and disease.

## Phagocytosis, phagocytic killing, and neutrophil extracellular traps

Phagocytosis is a specialized form of receptor-mediated endocytosis that is utilized by neutrophils and macrophages to ingest particles and microbes that are at least 0.5 microns in size^1^. The list of phagocytic receptors continues to grow and includes molecules that bind directly to microbes such CEACAMs, lectins, and integrins as well as receptors that engage microbes opsonized with IgG or complement fragments^2^. In all cases, signaling downstream of these receptors triggers local actin polymerization that is essential for extension of membrane pseudopodia around the microbe and its internalization.

A distinguishing feature of neutrophils is the fact that phagocytic receptor signaling also elicits rapid deployment of oxidative and non-oxidative host defense mechanisms via simultaneous assembly and activation of the Nox2-containing NADPH oxidase complex and mobilization of specific and azurophilic granules. As a result, phagosome–granule fusion and the production of superoxide anions and other toxic ROS is typically apparent within 60 seconds of particle or microbe binding^3–5^. The speed and efficiency of this process creates a highly lethal milieu in the phagosome lumen that consists of oxidants, cationic antimicrobial peptides, iron binding proteins, and enzymes such as elastase and myeloperoxidase (MPO). The formation of such an environment is in keeping with the ability of neutrophils to kill the majority of ingested microbes within 30 minutes^6–8^. By contrast, phagosome maturation in macrophages is relatively slow and consists of incremental modification of nascent phagosomes via sequential interaction with early endosomes, late endosomes, and lysosomes^9^.

The importance of NADPH oxidase-derived ROS to microbe killing is exemplified by the life-threatening infections that arise in patients who have inherited mutations in genes that encode subunits of this enzyme complex^10^. Pathogens that evade killing by neutrophils inhibit or evade toxic ROS and achieve this by inhibiting NADPH oxidase targeting, assembly, or activity^11–14^. Less is known about the mechanisms that control oxygen-independent defense mechanisms, including granule targeting and fusion, but recent data indicate that distinct Rab27-independent and -dependent mechanisms control azurophilic granule fusion with phagosomes and the plasma membrane, respectively^15^. Additional insight will likely be gleaned from further studies of pathogens that manipulate phagosome fusion with specific and/or azurophilic granules as part of their virulence strategies^2^.

The discovery of neutrophil extracellular traps (NETs) suggested the existence of an additional mechanism that allows neutrophils to trap and/or kill extracellular microbes^16^. In a landmark study, Brinkmann *et al*. reported that some activated neutrophils release decondensed chromatin fibers as web-like structures to which cationic neutrophil proteins such as elastase, histones, and MPO are bound^16^. These structures—aptly named NETs—can ensnare and kill microorganisms. Subsequent work by the same group of researchers reported a mechanism for the formation of NETs that requires ROS and, more notably, is a cytolytic process^17^. The formation of NETs that results from neutrophil lysis was proposed as a novel cell death program ultimately termed NETosis^17,18^. The formation of NETs and NETosis have often been used as synonymous terms for years, but whether the formation of NETs is always a result of the cytolytic process described as NETosis has been called into question^19,20^. Indeed, NETs can form from viable neutrophils^21–23^ or from neutrophils that have undergone non-specific lysis^24^. Thus, there is reasonable agreement in the field that the term “NETosis” should not be used to describe the formation of NETs unless a specific cell death mechanism is known^19,25,26^.

NETs have been studied extensively since the report by Brinkmann *et al*., and it is clear they can contribute to host defense. For example, Urban *et al*.^27^ and Branzk *et al*.^28^ reported that NETs are important for host defense against microbes that cannot be phagocytosed, such as fungi in hyphal form. More recently, Thanabalasuriar *et al*. demonstrated that NETs prevent dissemination of *Pseudomonas aeruginosa* from biofilms in a mouse infection model^29^. These roles for NETs in host defense are important and complementary to those of phagocytosis. However, the misconception that NETs are a primary means by which neutrophils kill microorganisms has become seemingly pervasive outside of the field of phagocyte biology. Here we compare and contrast phagocytosis and the formation of NETs (and outcomes associated with each process) as components of host defense.

## Physical sequestration of microbes following phagocytosis

There is no question that the ability of neutrophils to sequester microbes within an enclosed phagocytic vacuole (phagosome) is important for host defense. Ingested microbes can no longer disseminate freely, and molecules normally shed or secreted from pathogenic microbes are contained within phagosomes and thus unable to contribute to disease. By comparison, NETs ensnare microorganisms, but surface binding to a DNA scaffold is not likely to prevent dissemination fully. Moreover, NET-bound microbes are still able to release molecules such as bacterial endotoxins that can be detrimental. These latter points notwithstanding, studies by Branzk *et al*. and Thanabalasuriar *et al*. underscore the important role played by NETs if phagocytosis is not possible (because particles are too large). Branzk *et al*. also found that when phagocytosis occurs, it inhibits the formation of NETs via a mechanism involving sequestration of neutrophil elastase^28^. This intriguing finding provides support to the idea that phagocytosis and NETs have specific roles in host defense.

## High intraphagosomal concentration of microbicides

Few microorganisms have the ability to survive following phagocytosis by neutrophils. This is because intraphagosomal concentrations of neutrophil ROS and antimicrobial peptides are extraordinarily high, as intraphagosomal volume is limited (estimated at 1.2 µm^3^ for bacterial phagosomes)^30,31^. For example, Winterbourn *et al*. estimated the initial rate of superoxide generation as 5.2 mM/second and the intraphagosomal concentration of MPO as ~1 mM^31^. Based on these estimates, hypochlorous acid (HOCl), commonly known as the active ingredient in household bleach, is produced at 134 mM/minute. The granule protein concentration in the neutrophil phagosome has been estimated to be as high as 200 mg/mL^30^. Although MPO and elastase, components of neutrophil azurophilic granules, are hallmark features of NETs, the amount present on an extracellular DNA scaffold is likely far less than that present in phagosomes. Moreover, the concentration of ROS produced within phagosomes is simply not possible on or near NETs, as intact and viable neutrophils are needed to produce ROS, the half-life of these molecules is limited, and they diffuse readily. Therefore, compared with the microbicidal processes that occur within phagosomes (following phagocytosis), NETs have far less capacity to kill microorganisms.

## Nonphlogistic removal of apoptotic neutrophils

ROS and antimicrobial enzymes contained within neutrophil granules are not microbe specific and can also cause non-specific damage to host cells and tissues if they are released by exocytosis or following cell lysis^32–34^. Extracellular release of these toxic agents by neutrophils contributes significantly to many human inflammatory diseases. Therefore, it is not surprising that neutrophil lifespan and antimicrobial functions are highly regulated. Human neutrophils are terminally differentiated cells that undergo apoptosis constitutively at the end of their lifespan^35^. As neutrophils undergo apoptosis, they lose functional capacity, as indicated by defects in chemotaxis, phagocytosis, degranulation, and the ability to produce superoxide in response to certain stimuli^36^. Importantly, apoptotic neutrophils remain intact and are ingested by mononuclear phagocytes in a non-inflammatory process known as efferocytosis^35^. Removal of apoptotic neutrophils helps maintain immune system homeostasis and is key to resolution of the inflammatory response. This highly regulated process prevents host exposure to components of lysed neutrophils. Therefore, it could be argued that the formation of NETs by cytolysis is an incidental phenomenon that results from necrotic lysis rather than a unique type of cell death. This notion is gaining support among neutrophil biologists and cell death experts^19,25^.

## Noncytolytic versus cytolytic processes for the formation of NETs

The formation of NETs was described initially as a cytolytic process that culminates in the loss of plasma membrane integrity and release of cellular contents, most notably nuclear DNA, histones, and components of cytoplasmic granules, into the extracellular milieu^16,17^. Indeed, the majority of studies of NETs or their components either directly demonstrate or infer lysis of neutrophils. By comparison, Yousefi and colleagues first reported that extracellular traps form from mitochondrial DNA released from viable eosinophils^37^. This finding has since been extended to include neutrophils^21^ and basophils^38^. The formation of NETs from viable neutrophils is a noncytolytic process and is therefore compatible with the regulated turnover of neutrophils described above. More notably, such a process has the potential to function in concert with phagocytosis, as described above for microbes that cannot be ingested.

## NETs and human disease

Inasmuch as neutrophils are abundant leukocytes and have tremendous capacity for cytotoxicity, it is not unexpected that many diseases are associated with NETs. Although there are caveats with associating NETs and human disease, the sheer number of recent findings in this area underscores the importance of strictly regulated neutrophil activation and turnover (safe removal) to health and homeostasis. For example, NETs have been associated with multiple types of cancer and cancer metastasis^39,40^, including breast cancer^41^, hepatocellular carcinoma^42^, lung cancer^43^, ovarian cancer^44^, oral squamous cell carcinoma^45^, pancreatic cancer^46^, and thyroid cancer^47^. NETs have been implicated in autoimmune disorders such as lupus erythematosus^48–51^ and in the development of thrombosis^52–54^ and can contribute to the severity of sepsis^55,56^. Cell-free or extracellular DNA associated with elastase, MPO, and/or citrullinated histones has been associated with numerous respiratory diseases. NETs or NET components have been found in sputum from individuals with chronic obstructive pulmonary disease^57^ and severe asthma^58^, plasma from patients with severe influenza A virus infection^59^, bronchoalveolar lavage fluid from patients with ventilator-associated pneumonia^60^, and in plasma from patients with acute respiratory distress syndrome^61^. Most recently, NETs have been associated with severe COVID-19^62,63^.

Collectively, these data further support the idea that nonphlogistic turnover of neutrophils is essential to human health. In contrast to NETs, neutrophil phagocytosis is not associated directly with severe pathologies or disease.

## Conclusion

The idea that neutrophils utilize NETs extensively to eradicate microbes or that NETs are employed for host defense more prominently than phagocytosis has become increasingly pervasive outside of the field of phagocyte biology. Although a significant body of work supports the notion that NETs can contribute to host defense, the relative contribution of NETs to human host defense *in vivo* remains largely unknown. This issue is difficult to address, in part because the formation of NETs and neutrophil lysis can be synonymous. Identification of cell-free/extracellular DNA associated with citrullinated histones and/or neutrophil granule proteins is consistent with neutrophil lysis *in vivo*, a phenomenon linked to tissue destruction and disease. Indeed, the vast majority of studies published on the topic of NETs within the past two years underscore the role of NETs in disease. These data are consistent with the long-held tenet that uncontrolled release of neutrophil components, as occurs in cytolysis, is detrimental to health and exacerbates disease. Notably, the host immune system has evolved numerous tightly controlled mechanisms to prevent cytolysis, which implies that the formation of NETs via cell lysis is incidental, as is the contribution of such NETs to host defense.

Here we considered phagocytosis and NETs as components of host defense. Based on an assessment of historic and recently published studies and established paradigms, it seems clear that phagocytosis coupled to rapid phagosome–granule fusion and ROS production remains the primary means by which neutrophils eliminate invading microbes. More work is needed to determine the relative contribution of NETs and other lytic cell death mechanisms to host defense *in vivo*.

## References

[1] Griffin FM Jr Griffin JA Leider JE: Studies on the mechanism of phagocytosis. I. Requirements for circumferential attachment of particle-bound ligands to specific receptors on the macrophage plasma membrane. *J Exp Med.* 1975; 142(5): 1263–82. 10.1084/jem.142.5.1263 1194852 PMC2189973

[2] Allen LAH Criss AK: Cell intrinsic functions of neutrophils and their manipulation by pathogens. *Curr Opin Immunol.* 2019; 60: 124–9. 10.1016/j.coi.2019.05.004 31302568 PMC6800601

[3] Nordenfelt P Tapper H: Phagosome dynamics during phagocytosis by neutrophils. *J Leukoc Biol.* 2011; 90(2): 271–84. 10.1189/jlb.0810457 21504950

[4] Allen LA DeLeo FR Gallois A: Transient association of the nicotinamide adenine dinucleotide phosphate oxidase subunits p47*phox* and p67*phox* with phagosomes in neutrophils from patients with X-linked chronic granulomatous disease. *Blood.* 1999; 93(10): 3521–30. 10.1182/blood.V93.10.3521.410k21_3521_3530 10233905

[5] DeLeo FR Allen LAH Apicella M: NADPH oxidase activation and assembly during phagocytosis. *J Immunol.* 1999; 163(12): 6732–40. 10586071

[6] Segal AW: How neutrophils kill microbes. *Annu Rev Immunol.* 2005; 23: 197–223. 10.1146/annurev.immunol.23.021704.115653 15771570 PMC2092448

[7] Hampton MB Vissers MC Winterbourn CC: A single assay for measuring the rates of phagocytosis and bacterial killing by neutrophils. *J Leukoc Biol.* 1994; 55(2): 147–52. 10.1002/jlb.55.2.147 8301210

[8] Kobayashi SD Braughton KR Whitney AR: Bacterial pathogens modulate an apoptosis differentiation program in human neutrophils. *Proc Natl Acad Sci U S A.* 2003; 100(19): 10948–53. 10.1073/pnas.1833375100 12960399 PMC196908

[9] Flannagan RS Cosío G Grinstein S: Antimicrobial mechanisms of phagocytes and bacterial evasion strategies. *Nat Rev Microbiol.* 2009; 7(5): 355–66. 10.1038/nrmicro2128 19369951

[10] Nauseef WM Clark RA: Intersecting stories of the phagocyte NADPH oxidase and chronic granulomatous disease. *Methods Mol Biol.* 2019; 1982: 3–16. 10.1007/978-1-4939-9424-3_1 31172463

[11] Allen LAH McCaffrey RL: To activate or not to activate: Distinct strategies used by *Helicobacter pylori* and *Francisella tularensis* to modulate the NADPH oxidase and survive in human neutrophils. *Immunol Rev.* 2007; 219: 103–17. 10.1111/j.1600-065X.2007.00544.x 17850485

[12] IJdo JW Mueller AC: Neutrophil NADPH oxidase is reduced at the *Anaplasma phagocytophilum* phagosome. *Infect Immun.* 2004; 72(9): 5392–401. 10.1128/IAI.72.9.5392-5401.2004 15322037 PMC517486

[13] Edmisson JS Tian S Armstrong CL: *Filifactor alocis* modulates human neutrophil antimicrobial functional responses. *Cell Microbiol.* 2018; 20(6): e12829. 10.1111/cmi.12829 29377528 PMC5980721

[14] Smirnov A Daily KP Criss AK: Assembly of NADPH oxidase in human neutrophils is modulated by the opacity-associated protein expression state of *Neisseria gonorrhoeae*. *Infect Immun.* 2014; 82(3): 1036–44. 10.1128/IAI.00881-13 24343654 PMC3957997

[15] Johnson JL Brzezinska AA Tolmachova T: Rab27a and Rab27b regulate neutrophil azurophilic granule exocytosis and NADPH oxidase activity by independent mechanisms. *Traffic.* 2010; 11(4): 533–47. 10.1111/j.1600-0854.2009.01029.x 20028487 PMC2937183

[16] Brinkmann V Reichard U Goosmann C: Neutrophil extracellular traps kill bacteria. *Science.* 2004; 303(5663): 1532–5. 10.1126/science.1092385 15001782

[17] Fuchs TA Abed U Goosmann C: Novel cell death program leads to neutrophil extracellular traps. *J Cell Biol.* 2007; 176(2): 231–41. 10.1083/jcb.200606027 17210947 PMC2063942

[18] Steinberg BE Grinstein S: Unconventional roles of the NADPH oxidase: Signaling, ion homeostasis, and cell death. *Sci STKE.* 2007; 2007(379): pe11. 10.1126/stke.3792007pe11 17392241

[19] Yousefi S Stojkov D Germic N: Untangling "NETosis" from NETs. *Eur J Immunol.* 2019; 49(2): 221–7. 10.1002/eji.201747053 30629284

[20] Yipp BG Kubes P: NETosis: How vital is it? *Blood.* 2013; 122(16): 2784–94. 10.1182/blood-2013-04-457671 24009232

[21] Yousefi S Mihalache C Kozlowski E: Viable neutrophils release mitochondrial DNA to form neutrophil extracellular traps. *Cell Death Differ.* 2009; 16(11): 1438–44. 10.1038/cdd.2009.96 19609275

[22] Pilsczek FH Salina D Poon KKH: A novel mechanism of rapid nuclear neutrophil extracellular trap formation in response to *Staphylococcus aureus*. *J Immunol.* 2010; 185(12): 7413–25. 10.4049/jimmunol.1000675 21098229

[23] Yipp BG Petri B Salina D: Infection-induced NETosis is a dynamic process involving neutrophil multitasking *in vivo*. *Nat Med.* 2012; 18(9): 1386–93. 10.1038/nm.2847 22922410 PMC4529131

[24] Malachowa N Kobayashi SD Freedman B: *Staphylococcus aureus* leukotoxin GH promotes formation of neutrophil extracellular traps. *J Immunol.* 2013; 191(12): 6022–9. 10.4049/jimmunol.1301821 24190656 PMC3903389

[25] Boeltz S Amini P Anders HJ: To NET or not to NET:current opinions and state of the science regarding the formation of neutrophil extracellular traps. *Cell Death Differ.* 2019; 26(3): 395–408. 10.1038/s41418-018-0261-x 30622307 PMC6370810

[26] Galluzzi L Vitale I Aaronson SA: Molecular mechanisms of cell death: Recommendations of the Nomenclature Committee on Cell Death 2018. *Cell Death Differ.* 2018; 25(3): 486–541. 10.1038/s41418-017-0012-4 29362479 PMC5864239

[27] Urban CF Reichard U Brinkmann V: Neutrophil extracellular traps capture and kill *Candida albicans* yeast and hyphal forms. *Cell Microbiol.* 2006; 8(4): 668–76. 10.1111/j.1462-5822.2005.00659.x 16548892

[28] Branzk N Lubojemska A Hardison SE: Neutrophils sense microbe size and selectively release neutrophil extracellular traps in response to large pathogens. *Nat Immunol.* 2014; 15(11): 1017–25. 10.1038/ni.2987 25217981 PMC4236687

[29] Thanabalasuriar A Scott BNV Peiseler M: Neutrophil extracellular traps confine *Pseudomonas aeruginosa* occular biofilms and restrict brain invasion. *Cell Host Microbe.* 2019; 25(4): 526–536.e4. 10.1016/j.chom.2019.02.007 30930127 PMC7364305

[30] Winterbourn CC Kettle AJ: Redox reactions and microbial killing in the neutrophil phagosome. *Antioxid Redox Signal.* 2013; 18(6): 642–60. 10.1089/ars.2012.4827 22881869

[31] Winterbourn CC Hampton MB Livesey JH: Modeling the reactions of superoxide and myeloperoxidase in the neutrophil phagosome: *implications for microbial killing*. *J Biol Chem.* 2006; 281(52): 39860–9. 10.1074/jbc.M605898200 17074761

[32] Henson PM Johnston RB Jr: Tissue injury in inflammation. Oxidants, proteinases, and cationic proteins. *J Clin Invest.* 1987; 79(3): 669–74. 10.1172/JCI112869 3546374 PMC424175

[33] Brazil JC Parkos CA: Pathobiology of neutrophil-epithelial interactions. *Immunol Rev.* 2016; 273(1): 94–111. 10.1111/imr.12446 27558330 PMC5000857

[34] Weiss SJ: Tissue destruction by neutrophils. *N Engl J Med.* 1989; 320(6): 365–76. 10.1056/NEJM198902093200606 2536474

[35] Savill JS Wyllie AH Henson JE: Macrophage phagocytosis of aging neutrophils in inflammation. Programmed cell death in the neutrophil leads to its recognition by macrophages. *J Clin Invest.* 1989; 83(3): 865–75. 10.1172/JCI113970 2921324 PMC303760

[36] Whyte MK Meagher LC MacDermot J: Impairment of function in aging neutrophils is associated with apoptosis. *J Immunol.* 1993; 150(11): 5124–34. 8388425

[37] Yousefi S Gold JA Andina N: Catapult-like release of mitochondrial DNA by eosinophils contributes to antibacterial defense. *Nat Med.* 2008; 14(9): 949–53. 10.1038/nm.1855 18690244

[38] Morshed M Hlushchuk R Simon D: NADPH oxidase-independent formation of extracellular DNA traps by basophils. *J Immunol.* 2014; 192(11): 5314–23. 10.4049/jimmunol.1303418 24771850

[39] Grilz E Mauracher LM Posch F: Citrullinated histone H3, a biomarker for neutrophil extracellular trap formation, predicts the risk of mortality in patients with cancer *Br J Haematol.* 2019; 186(2): 311–20. 10.1111/bjh.15906 30968400 PMC6618331

[40] Rayes RF Mouhanna JG Nicolau I: Primary tumors induce neutrophil extracellular traps with targetable metastasis promoting effects. *JCI Insight.* 2019; 5(16): e128008. 10.1172/jci.insight.128008 31343990 PMC6777835

[41] Mouchemore KA Anderson RL Hamilton JA: Neutrophils, G-CSF and their contribution to breast cancer metastasis. *FEBS J.* 2018; 285(4): 665–79. 10.1111/febs.14206 28834401

[42] van der Windt DJ Sud V Zhang H: Neutrophil extracellular traps promote inflammation and development of hepatocellular carcinoma in nonalcoholic steatohepatitis. *Hepatology.* 2018; 68(4): 1347–60. 10.1002/hep.29914 29631332 PMC6173613

[43] Inoue M Nakashima R Enomoto M: Plasma redox imbalance caused by albumin oxidation promotes lung-predominant NETosis and pulmonary cancer metastasis. *Nat Commun.* 2018; 9(1): 5116. 10.1038/s41467-018-07550-x 30504805 PMC6269536

[44] Lee W Ko SY Mohamed MS: Neutrophils facilitate ovarian cancer premetastatic niche formation in the omentum. *J Exp Med.* 2019; 216(1): 176–94. 10.1084/jem.20181170 30567719 PMC6314534

[45] Li B Liu Y Hu T: Neutrophil extracellular traps enhance procoagulant activity in patients with oral squamous cell carcinoma. *J Cancer Res Clin Oncol.* 2019; 145(7): 1695–707. 10.1007/s00432-019-02922-2 31020419 PMC11810204

[46] Jung HS Gu J Kim JE: Cancer cell-induced neutrophil extracellular traps promote both hypercoagulability and cancer progression. *PLoS One.* 2019; 14(4): e0216055. 10.1371/journal.pone.0216055 31034495 PMC6488070

[47] Cristinziano L Modestino L Loffredo S: Anaplastic thyroid cancer cells induce the release of mitochondrial extracellular DNA traps by viable neutrophils. *J Immunol.* 2020; 204(5): 1362–72. 10.4049/jimmunol.1900543 31959732

[48] van der Linden M van den Hoogen LL Westerlaken GHA: Neutrophil extracellular trap release is associated with antinuclear antibodies in systemic lupus erythematosus and anti-phospholipid syndrome. *Rheumatology (Oxford).* 2018; 57(7): 1228–34. 10.1093/rheumatology/key067 29608758

[49] Liu Y Lightfoot YL Seto N: Peptidylarginine deiminases 2 and 4 modulate innate and adaptive immune responses in TLR-7-dependent lupus. *JCI Insight.* 2018; 3(23): e124729. 10.1172/jci.insight.124729 30518690 PMC6328098

[50] Odqvist L Jevnikar Z Riise R: Genetic variations in A20 DUB domain provide a genetic link to citrullination and neutrophil extracellular traps in systemic lupus erythematosus. *Ann Rheum Dis.* 2019; 78(10): 1363–70. 10.1136/annrheumdis-2019-215434 31300459 PMC6788882

[51] Mistry P Nakabo S O'Neil L: Transcriptomic, epigenetic, and functional analyses implicate neutrophil diversity in the pathogenesis of systemic lupus erythematosus. *Proc Natl Acad Sci U S A.* 2019; 116(50): 25222–8. 10.1073/pnas.1908576116 31754025 PMC6911190

[52] Nakazawa D Desai J Steiger S: Activated platelets induce MLKL-driven neutrophil necroptosis and release of neutrophil extracellular traps in venous thrombosis. *Cell Death Discov.* 2018; 4: 6. 10.1038/s41420-018-0073-2 30062055 PMC6060161

[53] Gollomp K Kim M Johnston I: Neutrophil accumulation and NET release contribute to thrombosis in HIT. *JCI Insight.* 2018; 3(18): e99445. 10.1172/jci.insight.99445 30232279 PMC6237233

[54] Perdomo J Leung HHL Ahmadi Z: Neutrophil activation and NETosis are the major drivers of thrombosis in heparin-induced thrombocytopenia. *Nat Commun.* 2019; 10(1): 1322. 10.1038/s41467-019-09160-7 30899022 PMC6428879

[55] Colón DF Wanderley CW Franchin M: Neutrophil extracellular traps (NETs) exacerbate severity of infant sepsis. *Crit Care.* 2019; 23(1): 113. 10.1186/s13054-019-2407-8 30961634 PMC6454713

[56] Barbosa da Cruz D Helms J Aquino LR: DNA-bound elastase of neutrophil extracellular traps degrades plasminogen, reduces plasmin formation, and decreases fibrinolysis: proof of concept in septic shock plasma. *FASEB J.* 2019; 33(12): 14270–80. 10.1096/fj.201901363RRR 31682515

[57] Dicker AJ Crichton ML Pumphrey EG: Neutrophil extracellular traps are associated with disease severity and microbiota diversity in patients with chronic obstructive pulmonary disease. *J Allergy Clin Immunol.* 2018; 141(1): 117–27. 10.1016/j.jaci.2017.04.022 28506850 PMC5751731

[58] Lachowicz-Scroggins ME Dunican EM Charbit AR: Extracellular DNA, neutrophil extracellular traps, and inflammasome activation in severe asthma. *Am J Respir Crit Care Med.* 2019; 199(9): 1076–85. 10.1164/rccm.201810-1869OC 30888839 PMC6515873

[59] Zhu L Liu L Zhang Y: High level of neutrophil extracellular traps correlates with poor prognosis of severe influenza A infection. *J Infect Dis.* 2018; 217(3): 428–37. 10.1093/infdis/jix475 29325098

[60] Mikacenic C Moore R Dmyterko V: Neutrophil extracellular traps (NETs) are increased in the alveolar spaces of patients with ventilator-associated pneumonia. *Crit Care.* 2018; 22(1): 358. 10.1186/s13054-018-2290-8 30587204 PMC6307268

[61] Lefrançais E Mallavia B Zhuo H: Maladaptive role of neutrophil extracellular traps in pathogen-induced lung injury. *JCI Insight.* 2018; 3(3): e98178. 10.1172/jci.insight.98178 29415887 PMC5821185

[62] Barnes BJ Adrover JM Baxter-Stoltzfus A: Targeting potential drivers of COVID-19: Neutrophil extracellular traps. *J Exp Med.* 2020; 217(6): e20200652. 10.1084/jem.20200652 32302401 PMC7161085

[63] Zuo Y Zuo M Yalavarthi S: Neutrophil extracellular traps and thrombosis in COVID-19. *medRxiv.* 2020; 2020.04.30.20086736. 10.1101/2020.04.30.20086736 33151461 PMC7642240

